# Vaccination with recombinant modified vaccinia virus Ankara prevents the onset of intestinal allergy in mice

**DOI:** 10.1186/2045-7022-3-S3-O24

**Published:** 2013-07-25

**Authors:** C Bohnen, A Wangorsch, S Schülke, M Burggraf, Y Suezer, A Schwantes, G Sutter, Z Waibler, G Reese, M Toda, S Scheurer, S Vieths

**Affiliations:** 1 Division of Allergology, Paul-Ehrlich-Institut, Langen, Germany; 2 Junior Research Group “Experimental Allergy Models”, Paul-Ehrlich-Institut, Langen, Germany; 3 Pr Research Group “Recombinant Measles Virus and Vaccines”, Paul-Ehrlich-Institut, Langen, Germany; 4 Institute for Infectious Diseases and Zoonosis, Ludwig-Maximilians-University, Munich, Germany; 5 Junior Research Group “Novel vaccination strategies and early immune responses”, Paul-Ehrlich-Institut, Langen, Germany

## Background

Modified vaccinia virus Ankara (MVA) encoding antigens are considered as safe vaccine candidates for various infectious diseases in humans. Here, we investigated the immune modulating properties of MVA encoding ovalbumin (MVA-OVA) on the allergen-specific immune response.

## Methods

The immune modulating properties of MVA-OVA were investigated using GM-CSF-derived BMDCs from C57BL/6 mice. OVA expression upon MVA-OVA infection of BMDCs was monitored by immunoblotting and ELISA. Activation and maturation markers on viable MVA-OVA infected mDCs were analyzed by flow cytometry. INF-g, IL-2, and IL-10 secretion was determined in a co-culture of BMDCs infected with wtMVA or MVA-OVA and MACS-sorted OVA-specific OT-I CD8^+^ and OT-II CD4^+^T cells. BALB/c mice were vaccinated with wtMVA, MVA-OVA or PBS, sensitized to OVA/alum and challenged with a diet containing chicken egg white. OVA-specific IgE, IgG1, and IgG2a and cytokine secretion from mesenteric lymph node (MLN)-cells were analyzed. Body weight, body temperature, food uptake and health condition of mice were monitored.

## Results

Infection with wtMVA and MVA-OVA induced comparable activation of mDCs. MVA-OVA infected BMDCs expressed OVA and induced enhanced IFN-g and IL-2 secretion from OVA-specific CD8^+^T cells in comparison to OVA, wtMVA or OVA plus wtMVA. Prophylactic vaccination with MVA-OVA significantly repressed OVA-specific IgE, whereas OVA-specific IgG2a was induced. MVA-OVA vaccination suppressed T_H_2 cytokine production in MLN cells and prevented the onset of allergic symptoms in a mouse model of OVA-induced intestinal allergy.

## Conclusion

MVA-OVA vaccination induces a strong OVA-specific T_H_1-immune response, likely mediated by the induction of IFN-g and IgG2a. MVA-based vaccines might be suitable for the treatment of allergic diseases.

## Disclosure of interest

None declared.

